# Partial resistance to clubroot in *Arabidopsis* is based on changes in the host primary metabolism and targeted cell division and expansion capacity

**DOI:** 10.1007/s10142-013-0312-9

**Published:** 2013-02-19

**Authors:** Mélanie Jubault, Christine Lariagon, Ludivine Taconnat, Jean-Pierre Renou, Antoine Gravot, Régine Delourme, Maria J. Manzanares-Dauleux

**Affiliations:** 1 Agrocampus Ouest, UMR1349 IGEPP, 35000 Rennes, France; 2 Université Européenne de Bretagne, Rennes, France; 3 INRA, UMR1349 IGEPP, 35653 Le Rheu, France; 4 UMR INRA 1165–CNRS 8114–UEVE, Unité de Recherche en Génomique Végétale, Université d’Evry-Val-d’Essone, CP 5708, 91057 Evry Cedex, France; 5 Université Rennes 1, UMR1349 IGEPP, 35000 Rennes, France; 6 Present Address: UMR IRHS, 42 rue Georges Morel, 49071 Beaucouzé Cedex, France; 7 UMR 1349 IGEPP INRA, Agrocampus Ouest Rennes, Université Rennes 1, BP35327, 35653 Le Rheu Cedex, France

**Keywords:** Clubroot, Quantitative resistance, *Arabidopsis*, *Plasmodiophora brassicae*, Microarray

## Abstract

To date, studies of the molecular basis of disease resistance mainly focused on qualitative resistance. However, deciphering mechanisms underlying quantitative resistance could lead to insights into the relationship between qualitative and quantitative resistance and guide the utilization of these two types of resistance to produce durably resistant cultivars. A functional genomics approach, using the CATMA whole-genome microarray, was used to detect changes in gene expression associated with partial quantitative resistance in the *Arabidopsis thaliana*–*Plasmodiophora brassicae* pathosystem. The time course of transcript abundance during partial clubroot resistance response was monitored at the whole plant level, and direct comparisons between partial resistance and susceptibility responses were made using the same host genotype. An increasingly complex host response was revealed, as was the differential influence of *P. brassicae* infection on the transcription of *Arabidopsis* genes according to the isolate used. We observed, at the transcriptomic level, that metabolic diversion by the pathogen was reduced or delayed, classical plant defense responses were induced earlier and/or more strongly, and cell enlargement and proliferation were actively inhibited in the partial quantitative resistance response compared to the susceptible one.

**Electronic supplementary material:**

The online version of this article (doi:10.1007/s10142-013-0312-9) contains supplementary material, which is available to authorized users.

## Introduction

Clubroot, caused by the obligate biotrophic protist *Plasmodiophora brassicae* Woron., is one of the most important diseases of *Brassica* crops, causing annual losses of 10–15 % worldwide (Dixon 2009). The life cycle of this soil-borne pathogen can be divided into two phases: a primary phase in which events are confined to the root hairs and a secondary phase that occurs in the cortex and the stele of the hypocotyl and roots of the infected plants. During the second phase, multinucleate plasmodia cause the hypertrophy (abnormal cell enlargement) and hyperplasia (uncontrolled cell division) of infected roots into characteristic clubs (Ingram and Tommerup 1972). These obstruct nutrient and water transport, stunt the growth of the plant, and consequently reduce crop yield and quality. Since the pathogen survives in the soil as resting spores readily transmittable and potentially viable for up to 15 years (Wallenhammar 1996), successful management of clubroot requires integrated control. The combination of adapted cropping practices as well as chemical and biological control methods is now a feasible strategy for the management of clubroot in *Brassica* (reviewed in Donald and Porter 2009); however, plant resistance is still the most powerful tool for combating clubroot disease (Diederichsen et al. 2009). Both qualitative and quantitative clubroot resistances were identified in different Brassicaceae species, including the three most economically important *Brassica* species: *Brassica napus*, *Brassica rapa*, and *Brassica oleracea* (reviewed in Piao et al. 2009). However, the commercial resistant cultivars from these species received primarily a single, dominant, and race-specific resistance gene, and several examples have now demonstrated the rapid adaptation of *P. brassicae* to widespread mono- or oligogenic clubroot resistance sources (Diederichsen et al. 2009). Although defined as a compatible host–pathogen interaction, partial resistance does limit the extent of the disease, either by rate-limiting pathogen multiplication or by reducing symptom severity. This form of resistance, frequently under polygenic control, is important for crop improvement and can be selected, often constituting an additional layer of resistance in the absence of R-mediated resistance and leading to high levels of phenotypic resistance (Poland et al. 2009). Furthermore, because it is controlled by multiple genes with small effects (leading to lower selection pressure on the pathogen) and/or is presumed to have a broader specificity, quantitative resistance should be overcome more slowly by the pathogen and appears to be an alternative for the development of durable host plant resistance (Boyd 2006; Brun et al. 2010; Palloix et al. 2009).

Up until now, the identification of components required for quantitative partial clubroot resistance was mainly based on quantitative trait loci mapping, both in cultivated species (Piao et al. 2009; Manzanares-Dauleux et al. 2000a, 2003; Rocherieux et al. 2004) and the model plant *Arabidopsis thaliana* (Jubault et al. 2008b). Functional studies on clubroot have mainly been conducted on the physiopathological mechanisms involved in the infection of susceptible hosts by *P. brassicae* (reviewed in Ludwig-Muller et al. 2009), but only a few studies, done on *Arabidopsis*, have focused on the mechanisms controlling quantitative resistance. Jubault et al. (2008a) reported strikingly different arginine catabolism signatures between susceptible and partially resistant plants. In particular, susceptible plants were characterized by a massive induction of arginase during the later stages of disease. This huge arginase induction actually constitutes a basal defense mechanism by reducing hormone-triggered cellular proliferation (Gravot et al. 2012). The lower induction of arginase in the partially resistant plants during *P. brassicae* infection reflects the attenuation or the delay of the pathogen influence on host metabolism in partially resistant plants compared to the situation in susceptible plants (Jubault et al. 2008a) and is more likely the result of partial clubroot resistance than its cause. Moreover, Gravot et al. (2011) showed that, although partial resistance to clubroot is not directly based on trehalose catabolism capacity, it is to some extent related to the tolerance to trehalose accumulation in the partially resistant accession Bur-0. However, the mechanisms underlying clubroot partial resistance currently remain largely unknown.

To gain further insight into this resistance type, a complementary approach is to identify candidate genes whose expression changes are in association with partial resistance, suggesting functional involvement. Using microarray technology, genome-wide information about patterns of gene expression during interactions between *Arabidopsis* or cultivated species and a variety of different pathogens was previously obtained. Analysis of host gene expression using microarrays provided significant insight into the transcriptional responses triggered during either R-mediated resistance (complete resistance) or basal defense (susceptibility; Tao et al. 2003; Marathe et al. 2004; Siemens et al. 2006; Jammes et al. 2005; Huibers et al. 2009; Radwan et al. 2011; AbuQamar et al. 2006; Swarbrick et al. 2008; Thilmony et al. 2006; Ditt et al. 2006; Agarwal et al. 2011; Mazarei et al. 2011). Microarray analyses were previously carried out to study *A. thaliana*–*P. brassicae* interactions; however, these were only done on disease development in the susceptible accession Columbia (Siemens et al. 2006; Agarwal et al. 2011).

Here, we report a large-scale gene expression profiling study of partial clubroot resistance in *A. thaliana* using the complete *Arabidopsis* transcriptome microarray (CATMA) chips (Crowe et al. 2003; Hilson et al. 2004). The Bur-0 accession is partially resistant to the isolate eH; however, it is fully susceptible to the isolate e_2_. This finding can thus be exploited to investigate, using the same host genotype, the transcriptional changes associated with these two levels of compatible interaction and determine specific molecular patterns associated with a partial resistance response compared to a susceptible one. At the transcriptomic level, the partial resistance response is associated with (1) a reduced or delayed host metabolic diversion by the pathogen, (2) an earlier and/or stronger induction of usual plant defense responses, and (3) a reduced expression of genes involved in cell enlargement and proliferation.

## Materials and methods

### Pathogen

The selection isolates eH and e_2_ (Fähling et al. 2003) belong to the *P. brassicae* pathotype P1, according to the host differential set established by Somé et al. (1996). They were kindly provided by J. Siemens (University of Dresden, Germany).

### Plant materials

Seeds from *Arabidopsis* accession Bur-0 (172AV) were obtained from the Versailles Resource Centre. This accession is partially resistant to the eH isolate (Alix et al. 2007) and susceptible to the e_2_ isolate, respectively. *B. napus* ssp. *oleifera* cv. “Nevin” (ECD6), *B. napus* ssp. *rapifera* cv. “Wilhelmsburger” (ECD10), and *B. napus* ssp. *oleifera* (Brutor), which constitute the host differential set established by Somé et al. (1996), and the highly clubroot susceptible *B. rapa* ssp. *pekiniensis* cv. “Granaat” (ECD5) were included as controls in each clubroot test.

### Experimental design and clubroot tests

**Fig. 1:**
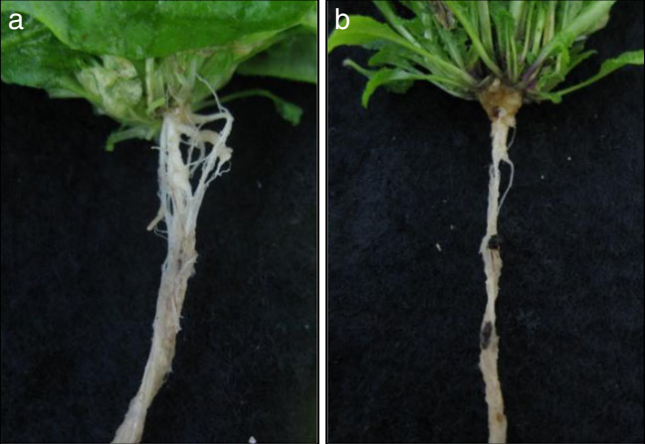
Typical symptoms during partial resistance and susceptibility responses to *P. brassicae* in *Arabidopsis*. **a** Tiny clubs are mainly confined to the secondary root system in the eH-inoculated plants (partial resistance), whereas the main and secondary root systems are replaced by a big club in the e_2_-inoculated plants (susceptible response) (**b**)

Two independent studies were performed (Fig. 1). In the first study, comparisons were made between control plants and plants inoculated with the eH isolate (comparisons 1–3). Inoculated and control plants were harvested at three time points: 1, 2, and 7 days post-inoculation (dpi; respectively stages 1.04 and 1.08; Boyes et al. 2001). The second study was performed only at 7 dpi, and comparisons were made between plants inoculated with either the eH or the e_2_ isolate (comparison 6). Each experiment was repeated twice.

*Arabidopsis* seeds were placed on wet blotting paper in Petri dishes at 4 °C for 3 days to synchronize germination; then, seeds were individually sown in 4-cm diameter pots containing a two thirds compost/one third vermiculite mix sterilized by autoclaving. *Arabidopsis* plants were grown under controlled environmental conditions (16-h light at 22 °C and 8-h dark at 19 °C) and inoculated 7 days after germination (stage 1.04; Boyes et al. 2001). The inoculum was prepared according to Manzanares-Dauleux et al. (2000a), and inoculation was performed by applying 1 mL of resting spore suspension (10^7^ spores per milliliter) to the crown of each seedling. The resting spore suspension was replaced by distilled water for the control plants. Thirty individual plants were collected per analysis point. Plants were thoroughly rinsed in different baths of water, frozen in liquid nitrogen, and stored at −80 °C until RNA isolation. To check that the inoculation was successful, clubroot susceptibility was evaluated in each test from 21 dpi (from stage 3.90 to 6.50; Boyes et al. 2001) and symptoms were recorded using the scale previously described for *B. oleracea* (Manzanares-Dauleux et al. 2000b): 0—no visible swelling; 1—very slight swelling usually confined to lateral roots; 2—moderate swelling on lateral roots and taproot; 2+—severe clubs on all roots, but some roots remain; 3—no root left, only one big gall. A disease index (DI) was calculated as described by Manzanares-Dauleux et al. (2000b): $\mathrm{DI}=\left( {{n_1}\times 25+{n_2}\times 50+{n_{{{2^{+}}}}}\times 75+{n_3}\times 100} \right)/N$, where *n*_*i*_ is the number of plants in the symptom class *i* and *N* the total number of plants tested. A line with a DI of zero is completely resistant and develops no clubroot symptoms, while a line with a DI of 100 is highly susceptible. Susceptibility to clubroot was also quantified by evaluation of the Ga/La pathological index [the ratio between gall area (Ga, in square centimeters) and rosette leaf area (roughly evaluated by the square of the longest leaf length—La, in square centimeters)] using image analysis, as previously described in Gravot et al. (2011).

### RNA isolation

For each analysis point, total RNA was extracted from approximately 30 mg of 30 pooled plants using the SV Total RNA Isolation kit (Promega, Madison, WI). Any remaining genomic DNA was removed by digestion with DNase I (DNA-free^TM^, Ambion®, Austin, TX). RNA integrity was checked with the Bioanalyzer from Agilent (Waldbroon, Germany).

### Microarray analyses

Microarray analyses were performed with the *A. thaliana* CATMA array containing 24,576 nuclear gene-specific tags (GST) corresponding to 22,089 nuclear genes, including 21,612 AGI-predicted genes and 477 Eugene-predicted genes (Allemeersch et al. 2005; Hilson et al. 2004). The GST (which are between 150 and 500 bp in length and show no more than 70 % identity with any other sequence in the genome) were spotted on UltraGAPS slides (Corning, NY) using a BioRobotics Microgrid II TAS spotter (Genomic Solution, Huntingdon, UK). Detailed information about CATMA and database access can be found online (Crowe et al. 2003).

**Fig. 2:**
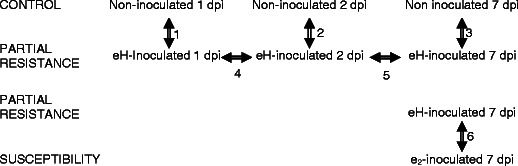
Experimental design for complete *Arabidopsis* transcriptome microarray (CATMA) transcript profiling of partial clubroot resistance

Six comparisons were performed during the time course analysis as described in Fig. 2. The array was hybridized simultaneously with cRNA from both samples labeled with Cy3 and Cy5 fluorescent dyes, respectively. For each comparison, a repeat was carried out using a second set of samples and a dye swap experiment to avoid dye bias and gene-specific dye bias (Martin-Magniette et al. 2005). Therefore, a total of 24 arrays were hybridized.

The complete microarray procedure has been fully described before (de Jong et al. 2006). Briefly, for each condition, mRNA from isolated RNA was amplified with the MessageAmp aRNA kit (Ambion, Austin, TX). Then, amplified mRNA was used as a template to synthesize modified cDNA with SuperScript II (Invitrogen, Carlsbad, CA) and random nonamers (Gene Link, Westchester County, NY) with the incorporation of cy3-dUTP and cy5-dUTP (NEN, Boston, MA; Puskas et al. 2002). Samples were combined, purified, and concentrated with YM30 Microcon columns (Millipore, Bedford, MA). The probes were hybridized overnight on CATMA arrays which were then scanned using a GenePix 4000A scanner (Axon Instruments, Foster City, CA). Spot intensities of the scans were determined by GenePix Pro 3.0 software (Axon Instruments).

### Statistical analysis of microarray data

Analysis of spot intensities from the CATMA arrays and applied statistics were performed as described previously (de Jong et al. 2006). Differentially expressed genes were selected based on a Bonferroni *p* value <5 %.

### Microarray data

Microarray data from this article were deposited at Array-Express (http://www.ebi.ac.uk/arrayexpress/; accession E-MEXP-363) and CATdb (http://urgv.evry.inra.fr/CATdb/; Project RA03-05_Clubroot**) according to the “Minimum Information About a Microarray Experiment” standards. Functional categories of differentially expressed genes were based on the Functional Catalogue (FunCat) scheme from the MatDB database (MIPS *A. thaliana* Database; http://mips.gsf.de/proj/funcatDB). Major metabolic pathways were analyzed using the MAPMAN software (Thimm et al. 2004).

### Real-time RT-PCR

First-strand cDNA was synthesized with Superscript™ II Reverse Transcriptase (Invitrogen) and oligo(dT)_15_ (Promega Corp.). For each gene, primers for real-time RT-PCR were designed on GSTs (Hilson et al. 2004) with Primer Express® v1.5 software (Applied Biosystems) and synthesized by Eurogentec. The genes, as well as the sequence of their specific oligonucleotides, are presented in Electronic supplementary material (ESM) Table 1. Duplicate quantitative assays were performed on 3 μL of 1/40 diluted cDNA using the SYBR-Green PCR Master kit (Applied Biosystems) with the ABI PRISM® 7700 Sequence Detection system (Applied Biosystems). To check the annealing specificity of each oligonucleotide, melting curve analysis (55–94 °C) was carried out at the end of amplification. For calculations, a standard curve was determined for each gene using different dilutions of the cDNA products. The expression levels for each target gene were then quantified following normalization to Actin8, the endogenous reference.

## Results

### Bur-0 is partially resistant to the eH isolate, but susceptible to the e_2_ isolate

The behavior of the Bur-0 accession was estimated from 21 dpi with *P. brassicae* isolates. As previously reported (Jubault et al. 2008b; Alix et al. 2007), the Bur-0 accession had an intermediate behavior in response to inoculation with the eH isolate, with a mean DI of 66 at 21 dpi. Bur-0 plants infected with this isolate typically showed only tiny clubs confined mainly to the secondary root systems and well-developed green rosettes (Fig. 1a and Table 1). On the contrary, Bur-0 was susceptible in response to inoculation with the e_2_ isolate, with a mean DI of 90 at 21 dpi. The plants infected with this isolate exhibited a big club replacing the main and secondary root systems and smaller rosettes (Fig. 1b and Table 1). A set of differential hosts, including susceptible and resistant genotypes of different *Brassica* species, was also evaluated at 49 dpi to characterize the isolate’s pathogenicity. This confirmed that both isolates, eH and e_2_ (Fähling et al. 2003), used in this study belong to the *P. brassicae* pathotype P1 (Somé et al. 1996).

**Table 1:** Evaluation of Bur-0 gall and leaf areas during partial resistance (inoculated with the eH isolate) and susceptibility (inoculated with the e_2_ isolate) responses to *P. brassicae*

Isolate	Gall area, Ga	Rosette leaf area, La	Ga/La pathological index
eH	0.06 ± 0.03	25.6 ± 1.76	13.63 ± 8.41
e_2_	0.13 ± 0.01	16.8 ± 1.02	49.09 ± 3.2

Means are estimated from 18 plants at 28 dpi. The Ga/La pathological index reflects the ratio between Ga and rosette La. Results are reported in square centimeters ± standard deviation

### Analysis of global changes in gene expression in partial resistance and susceptibility responses to *P. brassicae* infection

**Fig. 3:**
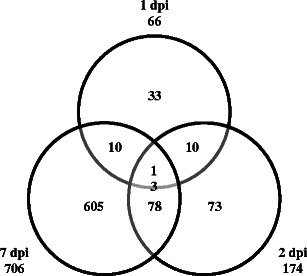
Venn diagram showing the differential distribution with time point of differentially expressed genes in eH-inoculated plants (partial resistance). *dpi* days post-inoculation

We know relatively little about changes in gene expression that occur during partial resistance response to *P. brassicae* infection and their specificity in comparison to the susceptible response. Thus, to gain insights into the transcriptional changes specifically associated with partial resistance, genome-wide expression analyses were carried out at the whole-plant level on the Bur-0 accession infected either with water, eH, or e_2_ isolates. First, to investigate the timing and extent of transcriptional changes associated with partial clubroot resistance response, comparisons were made between the transcript profiles of Bur-0 plants inoculated with the isolate eH and water-inoculated plants (i.e., control plants) over the time course of infection. To specifically relate host responses to the pathogen life cycle, comparisons were made during the symptomless phase at 1, 2 (corresponding to the primary phase), and 7 dpi (corresponding to the initiation of secondary infection in the cortex; Mithen and Magrath 1992; Puzio et al. 2000; Devos et al. 2006; Fig. 2, comparisons 1–3). Statistical analysis of these comparisons revealed that 822 genes were significantly differentially expressed. Thus, 4 % of the 22,089 *Arabidopsis* genes represented on the CATMA chip displayed changes in mRNA levels in the plants inoculated with the eH isolate. Among the 822 differentially expressed genes in partial resistance response to *P. brassicae*, 329 were up-regulated whereas 483 were down-regulated. In addition, the expression of ten genes was initially induced, but was then down-regulated (or vice versa) during the time course. A Venn diagram of comparisons 1–3 (Fig. 3) showed that the pattern of host gene expression in partial resistance response became increasingly complex over the time course, with 66, 174, and 706 genes differentially expressed at 1, 2, and 7 dpi, respectively.

In order to identify specific transcript changes between partial resistance and susceptibility responses, the transcript profiles of Bur-0 plants inoculated with either the eH isolate (leading to partial resistance) or with the e_2_ isolate (leading to complete susceptibility) were compared. Comparisons between the non-inoculated and eH-inoculated plants (described above) showed that host response at the whole-plant level was mainly induced at 7 dpi, which corresponds to the initiation of secondary infection in the cortex. Thus, we chose this kinetic point to make comparisons between responses to the eH and e_2_ isolates (Fig. 2, comparison 6). Statistical analysis of this comparison revealed 210 genes displaying significant differential expression at 7 dpi. Thus, only 1 % of the 22,089 *Arabidopsis* genes represented on the CATMA chip displayed changes in mRNA levels between susceptible and partial resistance responses. Among these, 120 genes were expressed at a higher level in eH-inoculated plants compared to the e_2_-inoculated ones, whereas 90 genes were expressed at a lower level. Only 94 genes showing differential expression were common to both experiments (comparison between non-inoculated and eH-inoculated plants and comparison between eH- and e_2_-inoculated plants).

**Fig. 4:**
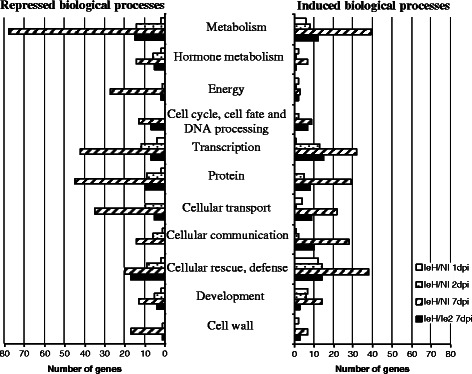
Distribution of the differentially expressed genes classified into functional categories. Genes were assigned to functional categories based on the Functional Catalogue (FunCat) scheme in the MatDB database (MIPS *Arabidopsis thaliana* Database). Genes of unknown function are not shown. The number of genes identified by the Functional Catalogue is indicated on the *x*-axis

To investigate which biological processes the differentially regulated genes may be involved in, we classified genes according to the functional categories defined by the Functional Catalogue (FunCat) in the MatDB database (MIPS *A. thaliana* Database; Fig. 4). This functional categorization carried out on all the genes identified in the time course comparisons between Bur-0 eH-inoculated and control plants showed that genes belonging to all functional groups were affected during the increasing host response to *P. brassicae* infection in the partial resistance response. Furthermore, for almost all biological processes, genes were more often seen to be repressed than induced, with the exception of genes involved in cellular rescue, defense, and cellular communication which were induced. Functional categorization carried out on the genes identified in comparisons between eH- and e_2_-inoculated plants showed that most of these genes are involved in cellular rescue and defense, metabolism, and transcription-related processes (Fig. 4). A similar number of genes were expressed at a higher or lower level in almost all categories, except for cellular communication and hormone metabolism for which genes were predominantly up- and down-regulated, respectively.

### Differential expression in microarrays was confirmed by quantitative RT-PCR

**Fig. 5:**
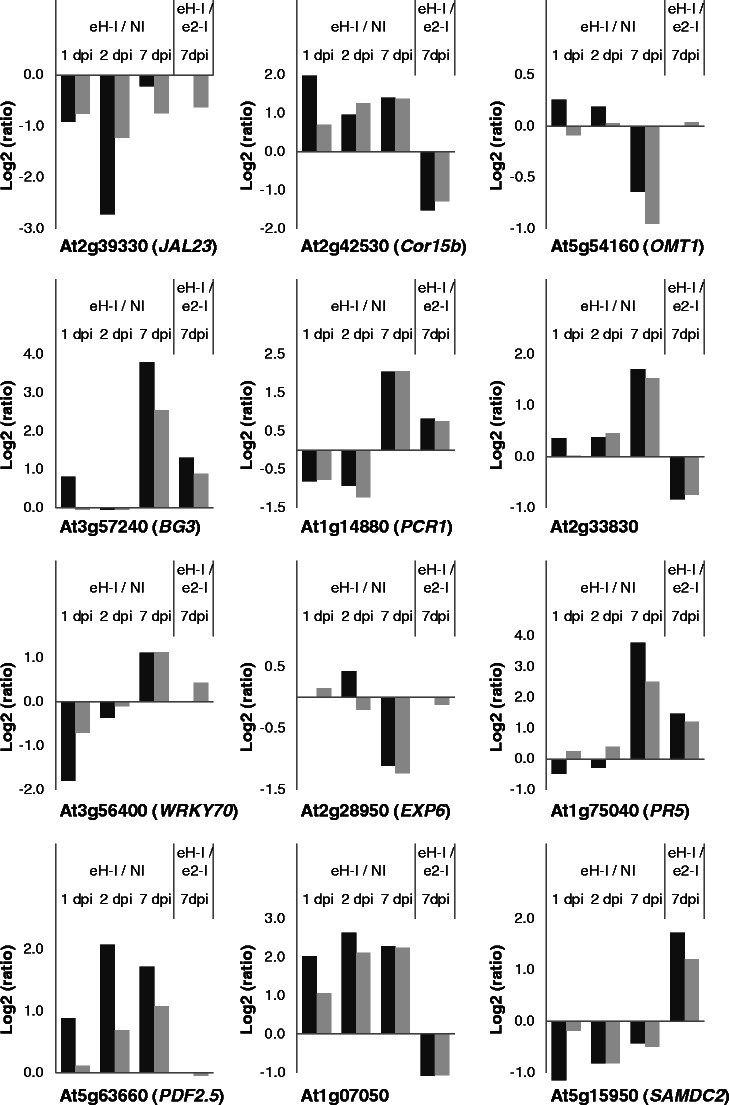
Validation of microarray data by quantitative RT-PCR. Microarray (*gray*) and quantitative RT-PCR (*black*) results are expressed in a log_2_ ratio obtained for comparisons between eH-inoculated (*eH-I*) and non-inoculated (*NI*) plants at 1, 2, and 7 dpi for the first experiment and between eH-inoculated (*eH-I*) and e_2_-inoculated (*e*_*2*_ *-I*) plants at 7 dpi for the second one. A positive ratio indicates that the gene is significantly induced in eH-inoculated plants in comparison to non-inoculated or e_2_-inoculated plants; a negative ratio means that the gene is significantly repressed in eH-inoculated plants in comparison to non-inoculated or e_2_-inoculated plants. *dpi* days post-inoculation

In order to validate the microarray data, a number of genes differentially expressed in at least one of the two studies were selected from different functional categories and their expression measured in control and infected tissues using quantitative RT-PCR. The RT-PCR profiles of these genes revealed that they exhibited the same temporal patterns and direction changes (up- or down-regulated) in gene expression as observed in the microarray experiments (Fig. 5).

### The molecular basis of clubroot partial resistance response

**Fig. 6:**
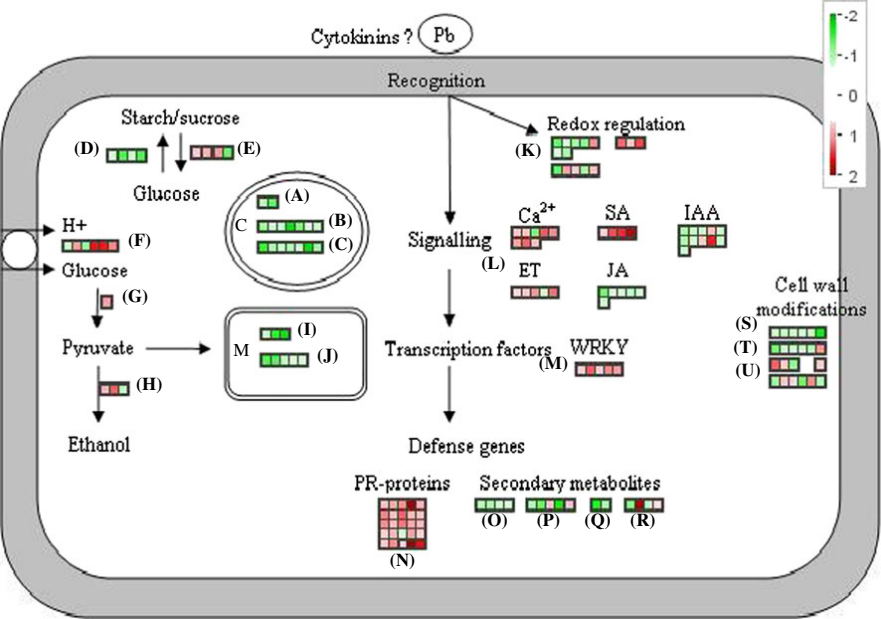
Schematic representation of the gene expression profiles of metabolic and defense pathways in eH-inoculated plants (partial resistance) at 7 days post-inoculation. Differential gene expression in tetrapyrrole synthesis (*A*), light reaction enzymes (*B*), Calvin cycle enzymes (*C*), starch and sucrose synthesis (*D*), starch and sucrose degradation (*E*), sugar transporters (*F*), glycolysis enzymes (*G*), fermentation enzymes (*H*), tricarboxylic acid cycle (*I*), electron transport enzymes (*J*), redox status regulation (*K*), signaling pathways (*L*), WRKY transcription factors (*M*), PR proteins (*N*), isoprenoid metabolism (*O*), lignin metabolism (*P*), wax metabolism (*Q*), flavonoid metabolism (*R*), cell adhesion (*S*), cell wall modification (*T*), and cell wall degradation (*U*). Each *square* symbolizes a differentially expressed gene. Numbers +2 to −2 on the color scale represent log_2_ of the fold change between the inoculated and non-inoculated plants. A positive ratio (*red square*) indicates that the gene is induced in eH-inoculated plants in comparison to non-inoculated plants; a negative ratio (*green square*) means that the gene is repressed in eH-inoculated plants in comparison to non-inoculated plants. *C* chloroplast, *M* mitochondrion, *Pb Plasmodiophora brassicae*

We then analyzed the role played by metabolic pathways using the MAPMAN software (Thimm et al. 2004; Fig. 6). MAPMAN is a user-driven tool that displays large data sets, such as gene expression data from *Arabidopsis* microarrays, onto diagrams of metabolic pathways or other processes in order to highlight general trends. Detailed numeric data are also presented in ESM Tables S2-B to S2-E.

#### Host’s primary metabolism regulation in response to eH infection

Major transcriptional changes in photosynthetic light reactions and carbohydrate metabolism occurred in the partial resistance response. Indeed, 22 % of all genes differentially expressed in the Bur-0 plants inoculated with the eH isolate were primary metabolism-related genes. The transcript levels of 18 genes involved in tetrapyrrole synthesis and photosynthesis (both the photochemical process and the Calvin cycle) were down-regulated mainly at 7 dpi (Fig. 6 and ESM Table S2-B). Probably linked to this decrease in photosynthesis, the transcript levels of seven genes involved in starch and sucrose synthesis and in the pentose phosphate pathway were also repressed at 7 dpi. In contrast, we found that genes coding starch-degrading enzymes, including *BAM3* (At4g20270) and *PWD* (At5g26570), and sucrose-degrading enzymes involved in the biosynthesis of hexose sugars and several sugar transporters were induced in infected plants at 7 dpi. In addition, the expression of genes involved in energy production was also altered. Several genes involved in the tricarboxylic acid cycle and in the respiratory chain were repressed. On the contrary, fermentation and production of ethanol appeared to be enhanced as genes encoding pyruvate decarboxylase *PDC1* (At5g54960) and *PDC2* (At4g33070) were up-regulated.

#### Transcription factor regulation in response to eH infection

The expression of many transcription factors and signaling components was altered in response to *P. brassicae* inoculation. Of the overall genes, 13 % were putative transcription factors differentially expressed mainly at 7 dpi. These transcription factors belong to several major families, including WRKY, MYB, Basic-Helix-Loop-Helix, Homeobox, and zinc-finger family proteins (Fig. 6 and ESM Table S2-C). Overall, the expression of transcription factors was down-regulated, with the exception of the WRKY class which was up-regulated in eH*-*inoculated plants. The fact that the transcription factors represent a wide distribution of gene families and that different expression profiles were observed may suggest that these are involved in controlling different processes and/or different phases of the response.

#### Host defense responses to eH infection

Further analysis showed that most of the 822 clubroot-induced *Arabidopsis* genes are potentially involved in host defense responses. In addition to WRKY transcription factors, some other signaling components, such as the calcium signaling pathway, were differentially expressed (Fig. 6 and ESM Table S2-D). Whereas two genes encoding calmodulins and calcium-binding proteins were down-regulated at the first two time points, seven were up-regulated at 7 dpi. Lastly, genes known or predicted to encode kinases were particularly abundant among the clubroot-induced genes. Numerous known or putative kinases were induced by *P. brassicae* inoculation at the end of the time course analysis. Of particular interest were 12 leucin-rich repeat (LRR)-containing receptor-like kinases. In contrast, four genes encoding G-proteins were down-regulated at 7 dpi.

The third largest functional class (12 %) contained genes involved in cell rescue and defense. Two thirds of these genes were up-regulated. At the first two time points post-inoculation, the defense-related group mainly included genes that were previously shown to be induced during abiotic stress, such as heat shock proteins or dehydrins. In contrast, at the later time point, the defense-related group was predominantly composed of genes encoding proteins frequently described during plant–pathogen interactions, such as enzymes involved in the removal of reactive oxygen species (ascorbate peroxidases, glutathione peroxidase, peroxidases, superoxide dismutase, glutathione-S-transferases) or pathogenesis-related (PR) proteins such as β-1,3-glucanases, chitinases, thaumatin, and defensins.

The transcript levels of some genes involved in secondary metabolism were also modulated in the partial resistance response. Two branches of the phenylpropanoid pathway were altered upon eH inoculation. In the flavonoid biosynthesis pathway, flavonol production appeared to have been favored since the gene encoding the flavonol synthase (At5g08640) was slightly up-regulated from 2 dpi. On the contrary, a gene encoding dihydroflavonol-4-reductase (At4g27250), which drives the same substrate (dihydroflavonol) into another pathway leading to anthocyanins and tannins, was down-regulated at 7 dpi. Among the flavonoid-related gene subset, *DMR6* (At5g24530) exhibited the highest induction level at 7 dpi. This gene, encoding an enzyme whose exact substrate is suspected to be a flavonoid, is known to be induced by the salicylic acid analogue BTH treatments and was reported to be involved in resistance to *Hyaloperonospora parasitica* (van Damme et al. 2008). Flavonoid accumulation was also reported in *Arabidopsis* roots following *P. brassicae* infection and was associated with the modulation of auxin efflux (Pasold et al. 2010). The lignin biosynthesis pathway was also affected; however, because genes involved in the same biosynthesis step showed conflicting differential regulations, the physiological impact of this pathway is unclear. Lastly, the expression of genes involved in isoprenoid and wax biosyntheses were down-regulated.

Analysis of the data also indicated that *P. brassicae* challenge significantly targeted several plant hormone signaling and stress response pathways. The down-regulation of genes encoding proteins involved in jasmonic acid (JA) biosynthesis, such as the lipoxygenase *LOX2* (At3g45140), in the JA signaling pathway, such as the desaturase *SSI2* (At2g43710), or jasmonate-inducible genes such as beta-glucosidase *BG1* (At1g52400), jacalin lectin proteins (At3g16450, At3g16470), hydroperoxide lyase *HPL1* (At4g15440), and the GH3 family member *JAR1* (At2g46370) suggested a down-regulation of the JA pathway at 2 and 7 dpi. The gene encoding *WRKY70* (At3g56400), a transcription factor which is well known to suppress jasmonic acid responses (Li et al. 2006), was up-regulated at 7 dpi. Interestingly, in contrast, three ethylene (ET)-related genes involved in either synthesis, such as 1-aminocyclopropane-1-carboxylic acid (ACC) synthase (At1g03400) and ACC oxidase (At5g43440), or response were up-regulated at 7 dpi.

Several genes related to the acid salicylic (SA) pathway were also induced, such as the isochorismate synthase *ICS1* (At1g74710) involved in SA biosynthesis and the lipase-like protein *PAD4* (At3g52430) and two *NPR1*-interacting proteins—*NIMIN1* (At1g02450) and *NIMIN2* (At3g25882)—components of this defense signaling pathway. Furthermore, the genes *SSI2* (At2g43710) and *JAR1* (At2g46370), previously shown to negatively interact with the SA pathway (Thatcher et al. 2005), were down-regulated.

#### Cell division and expansion regulation in response to eH infection

Several genes involved in growth and cell cycle control, including cellular organization and cell division, were also differentially expressed, mainly at 7 dpi (Fig. 6 and ESM Table S2-E). Furthermore, three members of fasciclin-like arabinogalactan proteins, involved in cell adhesion, were also repressed at 7 dpi. Numerous genes involved in cell wall modification were differentially expressed in the plants inoculated with the isolate eH, such as expansins and xyloglucan endotransglycosylases, which were down-regulated from 2 dpi. Pectinesterases were mainly up-regulated at 7 dpi. Genes involved in cell wall degradation such as β-1,4-endoglucanase, glycosyl hydrolases, polygalacturonase, and pectate lyase were also differentially expressed.

*P. brassicae* inoculation modulated the stress hormone response pathway (JA, ET, SA), but plant host physiology also experienced specific shifts in auxin and cytokinin responses as well. Twelve auxin-related genes were differentially expressed following infection by *P. brassicae* isolate eH. Several auxin-related genes were differentially expressed at 1 dpi, and their number increased with the infection. These include genes involved in auxin synthesis, such as nitrilase *NIT1* (At3g44310) and myrosinase-binding proteins (At2g39310, At2g39330), as well as auxin transport and response, and these were mostly down-regulated. Only three auxin-related genes were up-regulated at 7 dpi. Two genes involved in cytokinin metabolism were repressed at 7 dpi: the isopentenyltransferase *IPT9* (At5g20040), involved in cytokinin biosynthesis, and the response regulator *ARR4* (At1g10470).

### What are the differences between partial clubroot resistance and susceptibility responses?

#### Host’s primary metabolism regulation

Thirteen percent of all the genes that are differentially expressed between eH and e_2_ responses are predicted to function in primary metabolism. Almost all of them were found to be e_2_-specific or e_2_-enhanced regulations (ESM Table S2-B). As reported above, several genes involved in photosynthesis were repressed by eH at 7 dpi. Among those, the gene *GUN4* (At3g59400) involved in tetrapyrrole biosynthesis was found to be more repressed by e_2_ than by eH, and four other genes (At5g24120, At5g13630, At1g58290, and At2g21330) involved in tetrapyrrole biosynthesis, photosynthesis regulation, and Calvin cycle were specifically repressed by e_2_. Similarly, the e_2_-specific induction of *DPE* (At2g40840) and the e_2_-enhanced induction of *PWD* (At5g26570), both involved in starch degradation, may suggest that e_2_ infection could result in an enhanced starch degradation process. The level of induction of the gene encoding the pyruvate decarboxylase *PDC1* (At5g54960) was lower in the eH response, suggesting that the metabolic shift from aerobic to anaerobic fermentation associated with *P. brassicae* inoculation reported in the first microarray comparisons was also less pronounced in the partial resistant response than in the susceptible one.

#### Transcription factor regulation

Several putative transcription factors, representing almost 11 % of the differentially expressed genes, showed differential expression depending on response type. Seven transcription factors belonging to the WRKY, MYB, bZIP, and zinc finger families were specifically repressed by e_2_ infection, and two were specifically induced by eH infection (WRK46 and At5g10380 coding a C3HC4-type RING finger protein).

#### Host defense responses

Compared to the gene expression patterns observed in response to eH, defense responses upon inoculation with the more aggressive isolate e_2_ were lower. Six percent of the genes which were differentially expressed between eH- and e_2_-inoculated plants are involved in signal transduction. Three genes coding kinases and three genes coding calmodulin-binding proteins were repressed specifically by infection with the isolate e_2_. Two genes coding protein kinase were also induced specifically by e_2_. One gene coding a calmodulin, one DUF receptor kinase, and one LRR-containing receptor kinase were specifically induced by eH. The second largest functional class (15 %) contained genes involved in cell rescue and defense. Approximately half of these genes were previously shown to be induced during abiotic stress and were mainly induced in the e_2_-inoculated plants. The others have been frequently described during plant–pathogen interactions, such as genes involved in the removal of reactive oxygen species (glutathione peroxidase, peroxidase, catalases, and glutathione-S-transferase) or genes encoding pathogenesis-related proteins (thaumatin and defensin). Few genes involved in secondary metabolism such as the phenylpropanoid and the isoprenoid biosynthesis pathways were differentially expressed between eH and e_2_ responses. Two genes involved in the phenylpropanoid pathway displayed e_2_-specific or e_2_-enhanced induction. The transcript levels of the genes At1g06570 and At4g32770 encoding 4-hydroxyphenylpyruvate dioxygenase and tocopherol cyclase, respectively, were specifically repressed in response to e_2_, suggesting that this regulation might be involved in susceptibility. Lastly, the repression of the *CER1* gene (At1g022050) involved in wax biosynthesis was enhanced in response to eH, suggesting that this regulation could be involved in partial resistance.

At 7 dpi, several JA-related genes were found to be specifically induced by e_2_ (ESM Table S2-D and Fig. S3), such as the JA biosynthetic genes *LOX2* (At3g45140), encoding lipoxygenase, and AOS (At5g46250), encoding allene oxide synthase, and the JA-inducible genes *BG1* (At1g52400) encoding a beta-glucosidase and CYP81D1 (At3g28740). The induction of the SA pathway appeared to be enhanced in eH response as the lipase-like protein *PAD4* (At3g52430) and the *NPR1*-interacting protein NIMIN2 (At3g25882) inductions were found to be clearly specific or enhanced in response to eH. e_2_-specific repression, at the transcriptional level of few ethylene-related genes in at least one replicate (At5g25190, At5g61590, At5g47220, and At2g27050) may suggest also an enhanced ET pathway in eH response.

#### Cell division and expansion regulation

Four genes involved in cell growth and cycle control display e_2_-specific or e_2_-enhanced induction: the annexin *ANN4* (At2g38750), the expansin *EXP16* (At3g55500), a caldesmon-related protein (At1g52410), and a nodulin mtN3 family protein (At5g23660). The genes At4g02330 encoding a putative pectinesterase and *FLA9* (At1g03870) encoding a fasciclin-like arabinogalactan protein were specifically repressed in the e_2_ response. The gene *SEN4* (At4g30270) encoding an endo-xyloglucan endo1,4-*β* glucanase was more repressed by e_2_ than by eH.

Lastly, several auxin-related genes were differentially affected by eH and e_2_ isolates in at least one replicate. These include genes involved in auxin biosynthesis, such as nitrilase *NIT1* (At3g44310) and myrosinase-binding proteins (At1g52000, At2g39330), which were more repressed in response to eH than to e_2_. Genes involved in auxin response (At1g16510, At2g33830) were also repressed in response to the eH isolate. In particular, the gene At2g33830 encoding a dormancy/auxin-associated protein, previously reported as induced by eH inoculation, showed lower expression in response to eH than to e_2_.

## Discussion

The identification of genes regulated in partial clubroot resistance responses represents a major challenge for understanding the basis of partial quantitative resistance. Genome-wide comparative transcriptional analyses revealed here major differential gene expressions including a reduced or delayed metabolic diversion by the pathogen, an earlier and/or stronger induction of classical defense responses, and an active inhibition of cell enlargement and proliferation in the clubroot partial resistance response compared to the susceptible one.

A relatively small proportion of genes displayed significant changes in expression during the partial resistance response to the *P. brassicae* isolate eH. This result is in agreement with the earlier transcriptomics or proteomics works carried out at the early stages of host–pathogen interaction on fully susceptible Brassicaceae accessions (Agarwal et al. 2011; Devos et al. 2006; Cao et al. 2008) and contrasts with the results of Siemens et al. (2006) who reported high numbers of differentially expressed genes at greater fold change during the second stage of the disease. Together, these results suggest that during the asymptomatic phase of the *P. brassicae* life cycle, fewer morphological and physiological changes occur in the host compared with the secondary stage, when the host roots exhibit growing galls. Expression pattern comparisons between non-inoculated and eH-inoculated plants revealed an initial host response from 1 dpi that became increasingly complex. Relatively few genes were differentially expressed at 1 and 2 dpi, during the first contact between primary zoospores and root hairs and the development of primary plasmodia, and most were components of a general stress response. Most of the metabolic changes and defense systems specific to pathogen response were only triggered at 7 dpi, when secondary infection in the cortex is initiated. This increasing response pattern sharply contrasts with the decreasing host response to infection by *P. brassicae* observed in a susceptible *Arabidopsis* accession by Agarwal et al. (2011) and, thus, could be a key component of partial resistance.

### The pathogenesis process leads to reprogramming of the host’s primary metabolism

First, this study showed that upon inoculation with *P. brassicae*, the host’s primary metabolism underwent major reprogramming (Fig. 6), in particular with the repression of genes involved in the photochemical processes of photosynthesis and the Calvin cycle in infected plants, suggesting a low de novo carbohydrate production in leaves. This is rather coherent with the model proposed by Devos et al. (2006) where a leaf growth decrease coincides with the beginning of secondary infection. Together with the concomitant repression of genes involved in starch biosynthesis and the induction of genes involved in starch degradation and sugar transport (Fig. 6), those data are consistent with the accepted model where leaf carbon assimilates are reallocated to the infected root sink (Keen and Williams 1969; Evans and Scholes 1995). This flow toward the production of glucose was previously suggested in the proteome and transcriptome analyses of the susceptible ecotype Col-0 upon *P. brassicae* infection (Devos et al. 2006; Siemens et al. 2006). Furthermore, the suppression of invertase activity, which hydrolyzes sucrose into hexose monomers, using transgenic *Arabidopsis* lines leads to clearly reduced clubroot symptoms (Siemens et al. 2011).

Energy production mechanisms were also altered upon *P. brassicae* inoculation with the repression of cellular respiration processes and the induction of glycolysis and ethanol fermentation (Fig. 6). The infection appears to have induced a metabolic shift at 7 dpi from aerobic to anaerobic fermentation. This switch was also observed in the *A. thaliana*–*Agrobacterium tumefasciens* interaction (Deeken et al. 2006). As a result of diffusional limitations due to gall formation, cells in the infected tissues may easily become hypoxic and switch to fermentative energy metabolism. A second hypothesis was suggested by Koch et al. (2000) who showed that alcohol dehydrogenase *ADH1*, a main regulatory enzyme of ethanol fermentation, responds to sugars at physiological concentrations in fully oxygenated maize root tips. The accumulation of sugars at the infection sites might thus induce a hypoxia-like response and consequently force plant cells to switch to fermentative energy metabolism.

These metabolic changes occurred in both susceptible and partial resistance responses. However, several gene regulations related to photosynthesis and starch degradation suggest that the consequences on host primary metabolism were more dramatic in the susceptible response than in the partial resistance one. Consistent with these observations, Wagner et al. (2012) showed in *B. napus* that the primary metabolism of resistant genotypes was not much affected compared to susceptible ones in response to *P. brassicae* infection. Hence, the partial resistance response may be associated with a reduced or delayed metabolic diversion by the pathogen. However, we cannot conclude yet whether this regulation constitutes the cause, by retarding plasmodia growth in host cells, or the result of partial clubroot resistance.

### Defense responses were induced earlier or at increased levels during the partial resistance response

The identification of genes differentially expressed in the partial resistance response to *P. brassicae* demonstrated the activation of defense responses common to the basal defense and *R*-mediated resistance responses (Hammond-Kosack and Parker 2003; Fig. 6). Moreover, a comparison of eH and e_2_ responses at 7 dpi showed that several genes involved in defense mechanisms specific to the pathogen infection response were activated or induced at a higher level in response to eH (i.e., partial resistance response). In response to the e_2_ isolate (i.e., susceptibility response), they were either not induced at all or induced at significantly lower levels. Instead, response to e_2_ included a specific or enhanced activation of the components of the general stress response, such as those observed in eH-inoculated plants at 1 and 2 dpi. In agreement with these results, both Agarwal et al. (2011) and Siemens et al. (2006) reported that during the infection of a susceptible *Arabidopsis* accession by *P. brassicae*, few defense and disease resistance responses were activated or were even strongly down-regulated. It is consequently tempting to speculate, by analogy to the proposed hypothesis for incompatible interactions, that stronger or earlier signaling events enable eH-inoculated plants to delay and/or attenuate the effects of virulence factors (Tao et al. 2003; Poland et al. 2009).

Following pathogen attack, early defense signaling events are amplified through the generation of secondary signaling molecules, such as SA, JA, and ET, which activate defenses both locally, at the site of infection, and systematically in non-infected tissues. We found here that both the SA and ET pathways were induced during the partial resistance response, whereas the JA pathway was repressed (Fig. 6). The involvement of the SA, JA, or the ET pathway in the Brassicaceae–*P. brassicae* pathosystem has been previously described. In Chinese cabbage, a highly susceptible host. JA levels increased during club development and may be involved in the up-regulation of nitrilase, myrosinase, and tryptophan oxidase, enzymes involved in IAA synthesis (Grsic et al. 1999). Variations in the concentration of ACC, the direct precursor of ET, were observed in infected Chinese cabbage roots during the initiation of secondary infection (Devos et al. 2005). Further evidence for the involvement of the ET and JA pathways in gall formation was also provided by mutant analyses (Siemens et al. 2002, 2006; Devos et al. 2006). In addition, transcriptomic analyses on the *Arabidopsis* susceptible accession Col-0 at the earlier and later stages of the infection showed the induction of the JA pathway and the repression of the SA and ET pathways (Siemens et al. 2006; Agarwal et al. 2011). Pretreatment of *Arabidopsis* plants with salicylic acid was able to reduce the susceptibility to *P. brassicae* (Agarwal et al. 2011). This is consistent with our results where repression of the JA pathway and induction of the ET and SA pathways were associated with a reduction in symptom severity.

### Partial resistance is associated with an inhibition of cell division and expansion

Lastly, we identified several genes which could be involved in clubroot-induced uncontrolled cell division and expansion, such as genes controlling cytoskeleton dynamics, cell adhesion, and cell wall modifications (Fig. 6). As expected, most of these genes were differentially expressed at 7 dpi, when secondary infection in the cortex is initiated. Furthermore, most expansins were also down-regulated at 7 dpi, which would consequently lead to reduced galls.

The intrinsic characteristics of clubroot disease point to an involvement of the plant hormones cytokinin and auxin. A role for cytokinins (Dekhuijzen and Overeem 1971; Devos et al. 2005; Muller and Hilgenberg 1986; Siemens et al. 2006) and auxins was established in gall development (Grsic et al. 1999; Ludwig-Muller et al. 1993, 1999, 2009; Neuhaus et al. 2000) as well as in the early stage of infection (Devos et al. 2006; Agarwal et al. 2011). The current study also supports a possible role for cytokinins in early infection events with the repression of cytokinin biosynthesis upon *P. brassicae* infection. Furthermore, consistent with our microarray results carried out on partial resistance response, transgenic plants with lower cytokinin levels were found to be more tolerant to clubroot (Siemens et al. 2006). Host auxin metabolism is redirected toward the synthesis of more auxin through the nitrilase pathway (Grsic-Rausch et al. 2000) as clubroot plasmodia act as a strong sink for auxins. Siemens et al. (2006) reported the expression induction of the genes encoding nitrilases 1 and 2 during club development in the susceptible accession Col-0. Moreover, Devos et al. (2006) showed, at the proteome level, the upregulation of myrosinase and myrosinase-binding protein at 4 dpi in the susceptible accession Col-0. In *B. rapa*, the level of expression of myrosinase increased in infected roots compared with controls (Grsic et al. 1999). In our microarray analysis, numerous genes involved in host auxin biosynthesis as well as auxin transport and response were down-regulated in eH-inoculated plants (Fig. 6). The down-regulation of this pathway is consistent with the reduced gall formation in the partial resistant response. In agreement with this result, a nitrilase 1 mutant *nit1* was shown to have reduced root gall size and lower free IAA content in clubs (Grsic-Rausch et al. 2000; Neuhaus et al. 2000), and transgenic plants reduced in nitrilase 2 showed slower development of root galls (Neuhaus et al. 2000). Consequently, differential regulation of metabolic pathways related to the development of clubroot symptoms in eH-inoculated plants is consistent with reduced gall formation and suggests the existence of an as yet unknown mechanism associated with the reduction of cell enlargement and proliferation in the partial resistance response.

Further work on these pathways, such as their genetic manipulation in *A. thaliana* transgenic plants or quantification of gene expression in a range of *Arabidopsis* and *Brassicas*, showing extreme and intermediate levels of resistance to clubroot, will provide insights into the mechanisms involved in partial clubroot resistance.

## Electronic supplementary material

Below is the link to the electronic supplementary material.

ESM 1 (PPT 180 kb)

ESM 2 (DOCX 12 kb)

ESM 3 (XLS 294 kb)
