# Supplementary material for: Partial resistance to clubroot in Arabidopsis is based on changes in the host primary metabolism and targeted cell division and expansion capacity
Source: Funct Integr Genomics. 2013 Feb 19;13(2):191–205. doi: 10.1007/s10142-013-0312-9 (PMC3664179; doi:10.1007/s10142-013-0312-9)
Supplement: Supplementary file 1 — (PPT 180 kb) [file 10142_2013_312_MOESM1_ESM.ppt]

## Slide 1
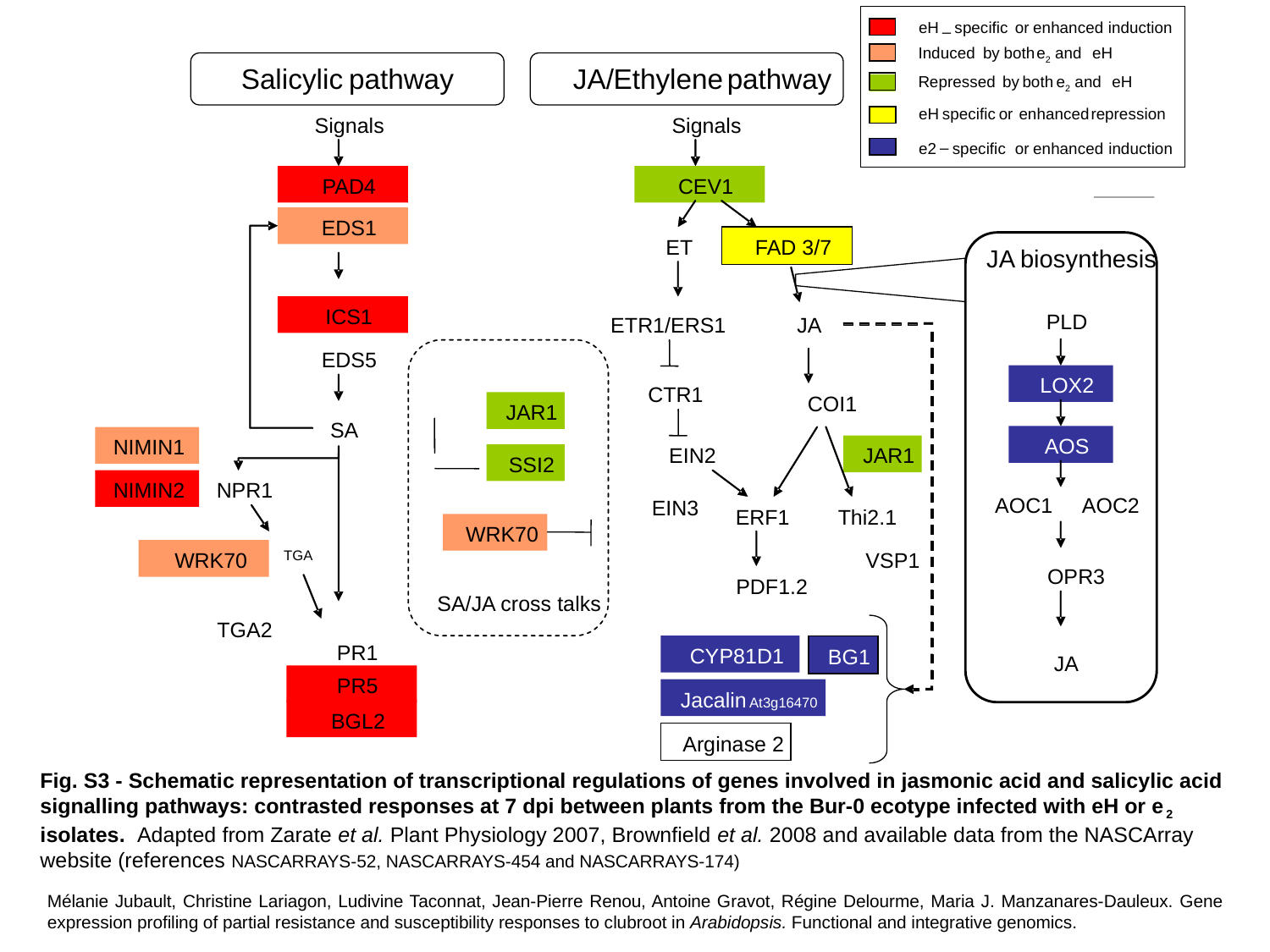

eH
specific
or
enhanced
induction
–
Induced
by
both
e2 and
eH
Salicylic
pathway
JA/
Ethylene
pathway
Repressed
by
both
e2 and
eH
eH
specific
 or
enhanced
repression
Signals
Signals
–
e2
specific
or
enhanced
induction
PAD4
CEV1
EDS1
ET
FAD 3/7
JA
biosynthesis
ICS1
PLD
ETR1/ERS1
JA
EDS5
LOX2
CTR1
COI1
JAR1
SA
AOS
NIMIN1
EIN2
JAR1
SSI2
NIMIN2
NPR1
AOC1
AOC2
EIN3
ERF1
Thi2.1
WRK70
TGA
WRK70
VSP1
OPR3
PDF1.2
SA/JA cross
talks
TGA2
PR1
CYP81D1
BG1
JA
PR5
Jacalin
At3g16470
BGL2
Arginase 2
Fig. S3 - Schematic representation of transcriptional regulations of genes involved in jasmonic acid and salicylic acid signalling pathways: contrasted responses at 7 dpi between plants from the Bur-0 ecotype infected with eH or e2 isolates. Adapted from Zarate et al. Plant Physiology 2007, Brownfield et al. 2008 and available data from the NASCArray website (references NASCARRAYS-52, NASCARRAYS-454 and NASCARRAYS-174)
Mélanie Jubault, Christine Lariagon, Ludivine Taconnat, Jean-Pierre Renou, Antoine Gravot, Régine Delourme, Maria J. Manzanares-Dauleux. Gene expression profiling of partial resistance and susceptibility responses to clubroot in Arabidopsis. Functional and integrative genomics.
