# Supplementary material for: Partial resistance to clubroot in Arabidopsis is based on changes in the host primary metabolism and targeted cell division and expansion capacity
Source: Funct Integr Genomics. 2013 Feb 19;13(2):191–205. doi: 10.1007/s10142-013-0312-9 (PMC3664179; doi:10.1007/s10142-013-0312-9)
Supplement: Supplementary file 2 — (DOCX 12 kb) [file 10142_2013_312_MOESM2_ESM.docx]

Mélanie Jubault, Christine Lariagon, Ludivine Taconnat, Jean-Pierre Renou, Antoine Gravot, Régine Delourme, Maria J. Manzanares-Dauleux. Gene expression profiling of partial resistance and susceptibility responses to clubroot in *Arabidopsis.* Functional and integrative genomics.

**Supplementary table S1 - Genes and oligonucleotides used in the real-time RT-PCR experiments.**

| **Loci** | **Description** | **Primers** | |
| --- | --- | --- | --- |
|  |  | **Forward primers** | **Reverse primers** |
| At1g49240 | *Actin8* | TTACCCGACGGACAAGTGATC | ATGATGGCTGGAAAAGGACTTC |
| At1g07050 | CONSTANS-like protein-related | TAAACGGAATTGTCCAGAGTTG | ATCGAGTACTGATCAAGAAGGCTCA |
| At2g42530 | Cold-responsive protein (*Cor15b*) | TCCTTTCTCAGTCACATAATCCAA | ATTTCGTGACGGATAAGACGAAG |
| At5g63660 | Plant defensin-fusion protein, putative (*PDF2.5*) | TGCCATAGTTTGCCGTAACG | AGTAGCAACGGCGATGGAAG |
| At3g57240 | Beta-1,3-glucanase (*BG3*) | TGAAGAGCCCTATATTGAACTGA | CCTTGAAGACACTTCACGATAAA |
| At1g75040 | Pathogenesis-related protein 5 (*PR-5*) | CTTCGAGTTAGATACAGTCGGGTTT | CAGCTGAGTGTAACAACTGACTAATA |
| At2g33830 | Dormancy/auxin associated family protein | GTGGAAATCAACGGTCACTAAC | GCTATACAGCGACGACACTAGG |
| At3g56400 | *WRKY70* | CCAGTTACGTCAATGGGAAAAC | CTCATTTTCGCTAAACTCGAAAT |
| At1g14880 | Expressed protein | CTTTATCGACGTTGTAATTTAACACT | CATGACCCGCTAAGATTGATT |
| At5g15950 | S-adenosylmethionine decarboxylase (*SAMDC2*) | TCTCCGAGATCTACCTTGAAATG | GATTCCCTATTCCTTCTCGTCCT |
| At5g54160 | 5-hydroxyferulic acid O-methyltransferase (*OMT1*) | CTAAACTTCCGACCAAAAATCCT | AGTTTACGGTTGGAGCAGGTTA |
| At2g28950 | Expansin, putative (*EXP6*) | GCAAGAATCCCAGGAGTTTACAAT | GCATCAGAGCCACCGTAGAAA |
| At2g39330 | Jacalin lectin family protein | AGTGCCATGTCGACTAAATGAAA | GAGCACACCAATTCTTATTGGAC |
